# A case report: Null-cell cardiac lymphoma in an English bulldog

**DOI:** 10.3389/fvets.2024.1256442

**Published:** 2024-02-07

**Authors:** Liza S. Köster, Kim Newkirk, Philip Krawec

**Affiliations:** ^1^Department of Small Animal Clinical Sciences, C247 Veterinary Medical Center, University of Tennessee College of Veterinary Medicine, Knoxville, TN, United States; ^2^Department of Biomedical Sciences and Diagnostic Sciences, University of Tennessee College of Veterinary Medicine, Knoxville, TN, United States

**Keywords:** cardiac tumor, echocardiogram, null-cell lymphoma, pericardial effusion, case report, right heart failure, dog

## Abstract

This case report describes a novel example of an extranodal null-type lymphoma in the myocardium of a middle-aged English bulldog who presented with signs of right heart failure. An echocardiogram found, in addition to the pericardial effusion, thickened right and left ventricular free walls and the interventricular septum. The right ventricular free wall myocardium had multinodular lesions, suspicious for infiltrative disease. The owner elected humane euthanasia, and permission for necropsy was obtained. Multifocal left and right ventricular nodules and an incidental aortic root mass were detected, the latter of which was later confirmed as a chemodectoma. Microscopically, the myocardial nodules were sheets of round cells consistent with a high-grade lymphoma. Neoplastic cells were not immunoreactive to CD3 (T-cell) or CD20 and CD79a (B-cell), Mum-1 (plasma cell), CD117 (mast cell), or CD18 (histiocyte). These findings are consistent with a high-grade, null-cell-type lymphoma.

## Introduction

Primary lymphoma of the myocardium and contiguous tissue (endo- and epicardium) is uncommon in dogs, accounting for 2 to 3% of primary cardiac neoplasms (1–3). It is one of the few cardiac neoplasms that is amenable to cytological diagnosis and potentially responsive to multiagent chemotherapy, emphasizing the importance of echocardiographic phenotype recognition (2). The diagnostic challenges of confirming cardiac neoplasia include the low sensitivity of echocardiography, which is even lower in the absence of pericardial effusion. Needle biopsy and endomyocardial biopsy of cardiac masses are invasive, and the cytological yield is highly variable. The test accuracy of predicting all types of cardiac neoplasia from pericardial effusion cytology is (1) generally considered poor (2%), with frequent false positives and negatives; (2) dependent on tumor type; and (3) better when the packed cell volume of the effusion is less than 10% (4). In contrast, the diagnostic utility of examining pericardial effusion in cardiac lymphoma has merit. In the largest report of canine cardiac lymphoma, 92% (11 of 12) of the dogs were diagnosed on the cytological examination of pericardial effusion (2).

Stage Vb lymphoma, according to the WHO classification scheme, refers to an extranodal site other than the spleen or liver, and the patient is clinically diagnosed with the disease (1). The definition of cardiac lymphoma in human literature is that the patient must present with symptoms of cardiac disease, usually right heart failure (RHF), with the bulk of the lymphoma intrapericardial in location, usually the right atrium, at the time of clinical diagnosis (5). This case report describes the presentation and imaging findings of a case of extranodal cardiac null-cell lymphoma, a rare form of lymphoma confirmed on necropsy in a middle-aged dog. Echocardiograms detected changes consistent with previously described cases of cardiac lymphoma in dogs. The differential diagnoses included neoplastic, infiltrative, inflammatory, infectious, and coagulation abnormalities. The diagnosis would likely have been confirmed on cytology if a pericardiocentesis was performed, but the owner elected euthanasia, resulting in necropsy and histopathologic confirmation.

## Case description

A 7-year-old female spayed English bulldog was referred to the University of Tennessee Veterinary Medical Center’s Emergency Service for the evaluation of increased respiratory rate and effort. Six days prior to presentation, the patient experienced an acute onset of hyporexia, along with tachypnea and increased respiratory effort. Thoracic radiographs obtained at another facility were concerning for aspiration pneumonia. Amoxicillin–clavulanate at a dose of 21 mg/kg orally every 12 h was instituted. The next day, the owners reported that the patient was clinically unchanged, prompting an evaluation by the primary veterinarian. The patient was administered injections of maropitant citrate (Cerenia-Zoetis Inc., USA) and cefovecin sodium (Convenia-Zoetis Inc., USA). The patient continued to show no improvement 2 days later, at which time an albuterol inhaler (90mcg, 1 actuation every 6–8 h as needed), a prescription diet (a/d canned-Hill’s Pet Nutrition, USA), and furosemide (2.3 mg/kg orally every 12 h) were dispensed. The respiratory symptoms persisted for 2 days; hence, the primary veterinarian prescribed metronidazole. The patient’s appetite remained poor, requiring syringe feeding of the previously prescribed diet. The respiratory rate and effort remained abnormal. This prompted a final evaluation at the primary veterinarian’s facility, where a single lateral thoracic radiograph showed evidence of pleural effusion, prompting referral for further diagnostics and therapy.

On presentation to the authors’ institution, the patient was quiet and alert. Non-invasive systolic blood pressure measured by Doppler ultrasound was 90 mmHg (normal, 90–159 mmHg). The patient’s heart rate was considered inappropriately high (174 beats per minute, normal 70–120 bpm) with tachypnea (60 breaths per minute [brpm], normal 17–34 brpm) and mildly increased respiratory effort, partially attributed to stress. Cardiopulmonary auscultation revealed slightly muffled heart and lung sounds. Stertor was noted, consistent with the patient’s confirmation. Abdominal palpation was limited due to body habitus. The remainder of the physical examination was unremarkable.

Oxygen saturation was appropriate with a SpO_2_ of 100% (reference >96%) (Radical-7 Pulse Oximeter-Masimo Corp., USA). Venous blood lactate (Lactate Plus-Nova Biomedical, USA) was minimally elevated (2.9 mmol/L, reference 0.5–2.0 mmol/L), attributed to mild volume depletion, poor cardiac output, or type B hyperlactatemia. Venous blood gas (Nova Stat Profile Prime Plus-Nova Biomedical, USA) revealed a mild, compensated metabolic alkalosis. Mild azotemia (BUN 49 mg/dL, reference 8–30; creatinine 1.9 mg/dL, reference 0.6–1.6), along with mild hyponatremia (136 mmol/L, reference 142–149.3) and hypochloremia (98 mmol/L, reference 113–118) were noted. These variations were attributed to pre-renal azotemia secondary to furosemide administration; alternatively, decreased renal blood flow and activation of the renin–angiotensin–aldosterone system were considered. No other electrolyte or acid–base disturbances were detected.

The patient underwent routine point-of-care ultrasound in a standardized manner (6). No free fluid was visualized in the abdomen. Evaluation of the thorax revealed moderate amounts of pleural effusion as well as a mild amount of pericardial effusion.

**Figure 1:**
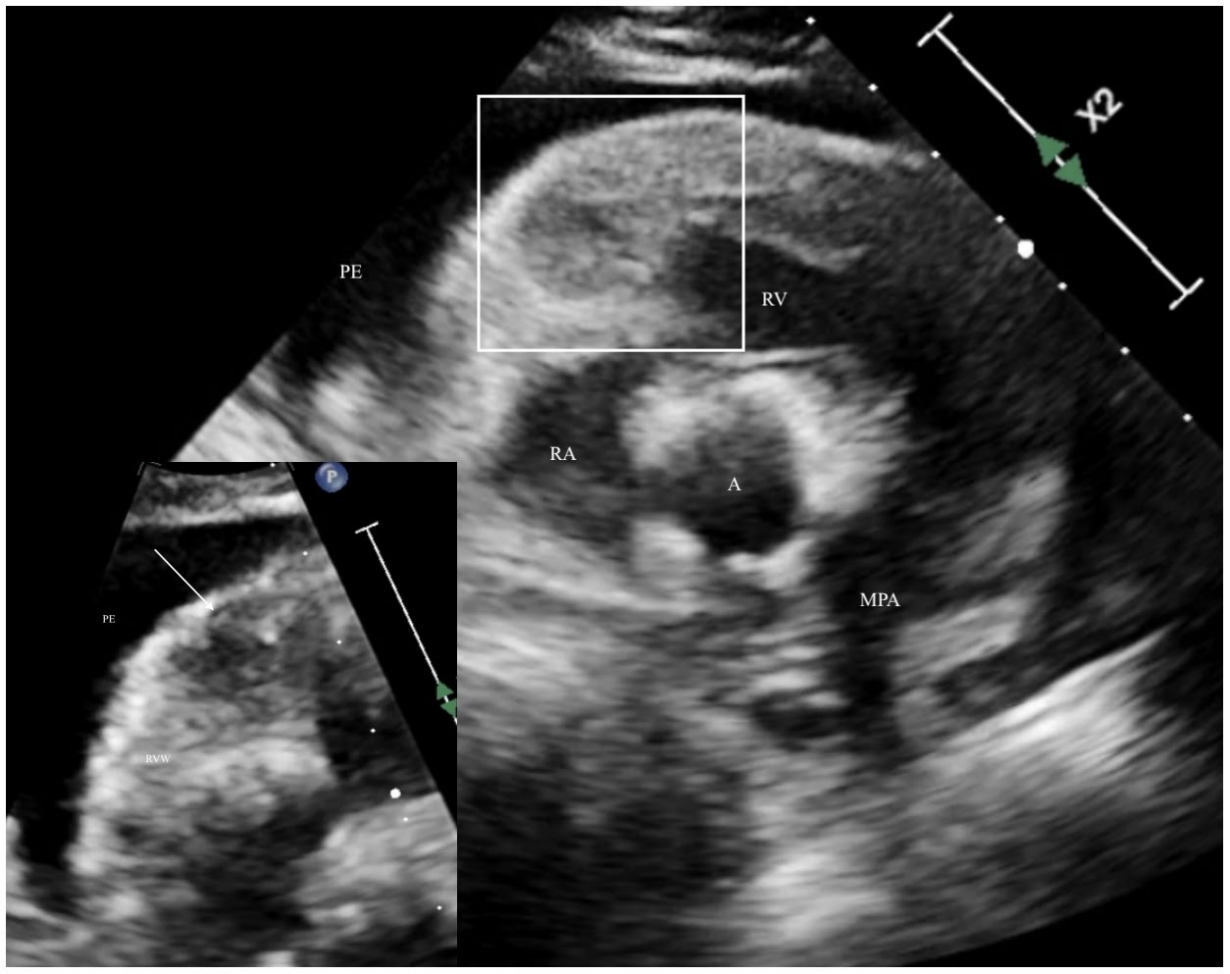
Echocardiogram optimizing the right ventricle with the right parasternal short axis view of the right ventricular outflow tract depicting irregular thickening and heterogenous echodensity surrounded by anechoic pericardial effusion. The white outline area is the location depicted in the zoomed insert on the lower left corner, a focused area of the right ventricular free wall base, which depicts the heterogenous echodensity of the myocardium with a hypoechoic, roughly spherical mass (arrow). A, aorta; MPA, main pulmonary artery; PE, pericardial effusion; RA, right atrium; RV, right ventricle; RVW, right ventricular wall.

After the owner’s consent, the patient underwent a complete echocardiogram (X5-1 probe and EPIQ 7 machine, Philips, USA), including a two-dimensional, M-mode, and Doppler examination as described in the canine (7). Ultrasound coupling gel over the left and right precordium was applied to allow an acoustic window; the fur was not clipped. A single-limb lead (lead II) was used for continuous electrocardiogram monitoring during the echocardiogram, which detected intermittent, monomorphic singlet ventricular premature complexes. On the right parasternal long-axis view, mild to moderate anechoic pericardial effusion (< 1 cm width) without evidence of tamponade was noted. The left ventricular free wall (1.9 cm, reference 0.6–1.2) and septum (1.5 cm, reference 6–1.2) were thickened when measured in diastole as compared to allometrically predicted reference intervals based on the dog’s body weight (8). A focal, roughly spherical-shaped, hypoechoic mass, measuring at least 1 by 1.08 cm in dimension visible on the right parasternal long axis (four chamber view), located within the anterior basilar right ventricular myocardium. There was diffusely irregular thickening of the anterior and septal right ventricular walls, which were hyperechoic and mottled in appearance due to heterogenous echogenicity (Figure 1), consistent with infiltration. The caudal vena cava and hepatic veins were judged to be dilated with absent distensibility during the respiratory cycle. The etiologies that were considered included the following: cardiac tumors, including lymphoma, myxosarcoma, hemangiosarcoma, sarcoma, and chemodectoma; infiltrative disease, including eosinophilic myocarditis and amyloidosis; bacterial or mycotic myocarditis; and organizing myocardial hematoma or thrombus. Recommendations for further investigation after the results of the echocardiogram were available that could be considered helpful in elucidating the etiology included pericardiocentesis and analysis of the effusion, including cytology, a complete peripheral blood count to investigate for an inflammatory leukogram or eosinophilia, serum biochemistry and fasted ionized calcium, and staging for potential primary or metastatic cardiac neoplasia, including imaging of the thorax and abdomen. No recommendations for managing the ventricular ectopy were made, as these were considered of low complexity and unlikely to have had a hemodynamic impact on the patient.

Given the infiltrative nature of the myocardial lesion and overall concerns for quality of life, the owners elected humane euthanasia. The patient was sedated with propofol (PropoFlo 28-Zoetis, USA) intravenously, followed by an intravenous injection of a pentobarbital solution (Fatal-Plus Solution-Vortech Pharmaceuticals Ltd., USA). The patient was submitted for a full necropsy.

**Figure 2:**
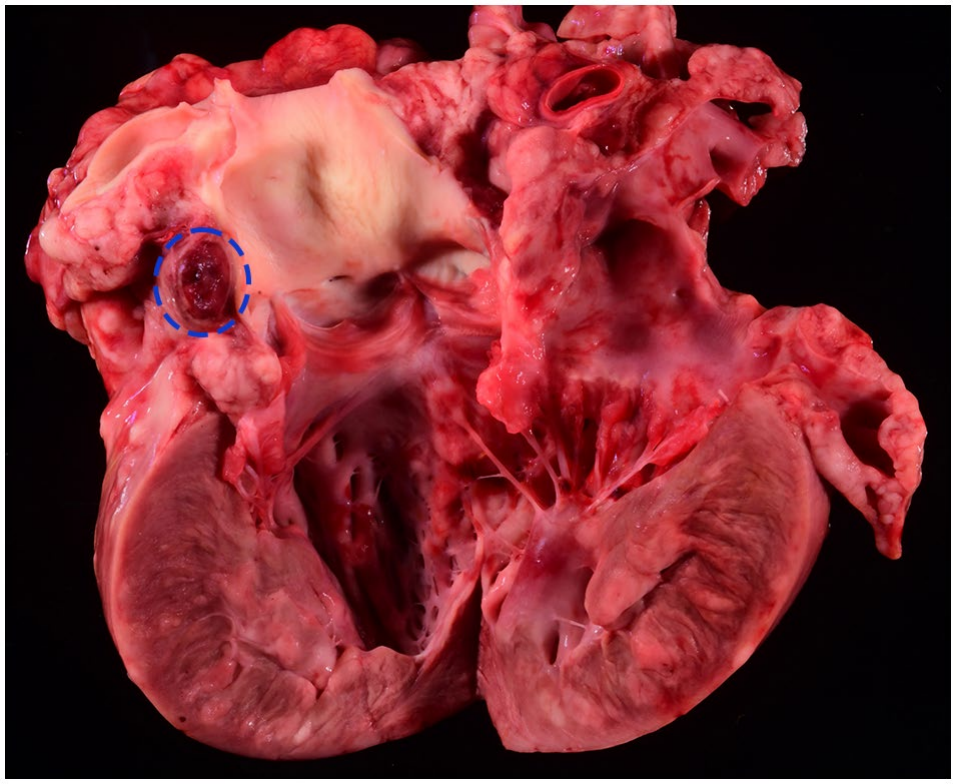
Gross section of the left ventricular outflow tract. Multifocal to coalescing, white to tan, soft nodules expand the myocardium; microscopically, these masses were composed of neoplastic round cells. Adjacent to the aorta root is an oval, red, well-circumscribed nodule (circled by a blue-dashed line), which is histologically consistent with a chemodectoma.

Necropsy confirmed that in addition to effusions in the pericardial and pleural spaces, there was a peritoneal effusion (1,000 mL in the thoracic cavity, 100–150 mL in the abdominal cavity, and 100 mL in the pericardial space); the fluid was clear and varied from light red in the thoracic cavity and pericardial space to dark red in the abdominal cavity. Throughout the thoracic cavity, there were multiple thin, friable, pale pink to tan strands of fibrin. The mediastinum was expanded by a clear, watery, and gelatinous material considered edema. The trachea contained clear, red mucoid material. The lung lobes were diffusely dark red to purple and collapsed, but all lung lobes would float in 10% formalin. The heart weighed 0.22 kg, which is 0.9% of body weight (adult normal is 0.7–1.2%). The myocardium of the right and left ventricular free walls was irregularly thickened by numerous multifocal to coalescing, white to tan, soft nodules ranging from 1 mm in diameter to 10x5x3mm (Figure 2). The liver weighed 0.72 kg, which was 3.03% of body weight (adult normal is 3–3.5%). The spleen and lymph nodes were grossly normal. Incidentally, mild hydrocephalus was detected.

At necropsy, heart, lung, brain, lymph node, liver, and kidney samples were collected and fixed in 10% neutral-buffered formalin and routinely processed for microscopic examination. Tissue sections were stained with hematoxylin and eosin and examined microscopically. There were no significant findings in the lung, brain, lymph node, liver, or kidney. In heart sections, the myocardium of the left and right ventricular-free walls and the aortic outflow tract were multifocally expanded by round cells arranged in sheets. The round cells had distinct cell borders, small amounts of eosinophilic cytoplasm, and round nuclei. The nuclei were about the size of an erythrocyte, and there were 108 mitotic figures in ten 400x (2.37 mm^2^) fields, with occasional bizarre mitoses. Anisokaryosis was mild. There were multifocal areas where neoplastic cells were necrotic. Cardiac myocytes were occasionally entrapped within the neoplastic cells and were small (atrophy) or fragmented. Given the lack of similar changes in other organs, the cardiac infiltrates were consistent with a primary cardiac lymphoma, which was further characterized as small cells and high grade.

Adjacent to the aorta, there was another distinct population of neoplastic cells. These neoplastic cells were polygonal, arranged in nests and packets, and supported by a thin fibrovascular stroma. Cells had moderate amounts of basophilic granular cytoplasm and round nuclei. Anisokaryosis was mild, and mitoses were absent. A Churukian–Schenk stain highlighted cytoplasmic granules of polygonal neoplastic cells brown to black. The stain did not highlight any granules in the previously described neoplastic round-cell population. These findings were consistent with an aortic body chemodectoma.

**Figure 3:**
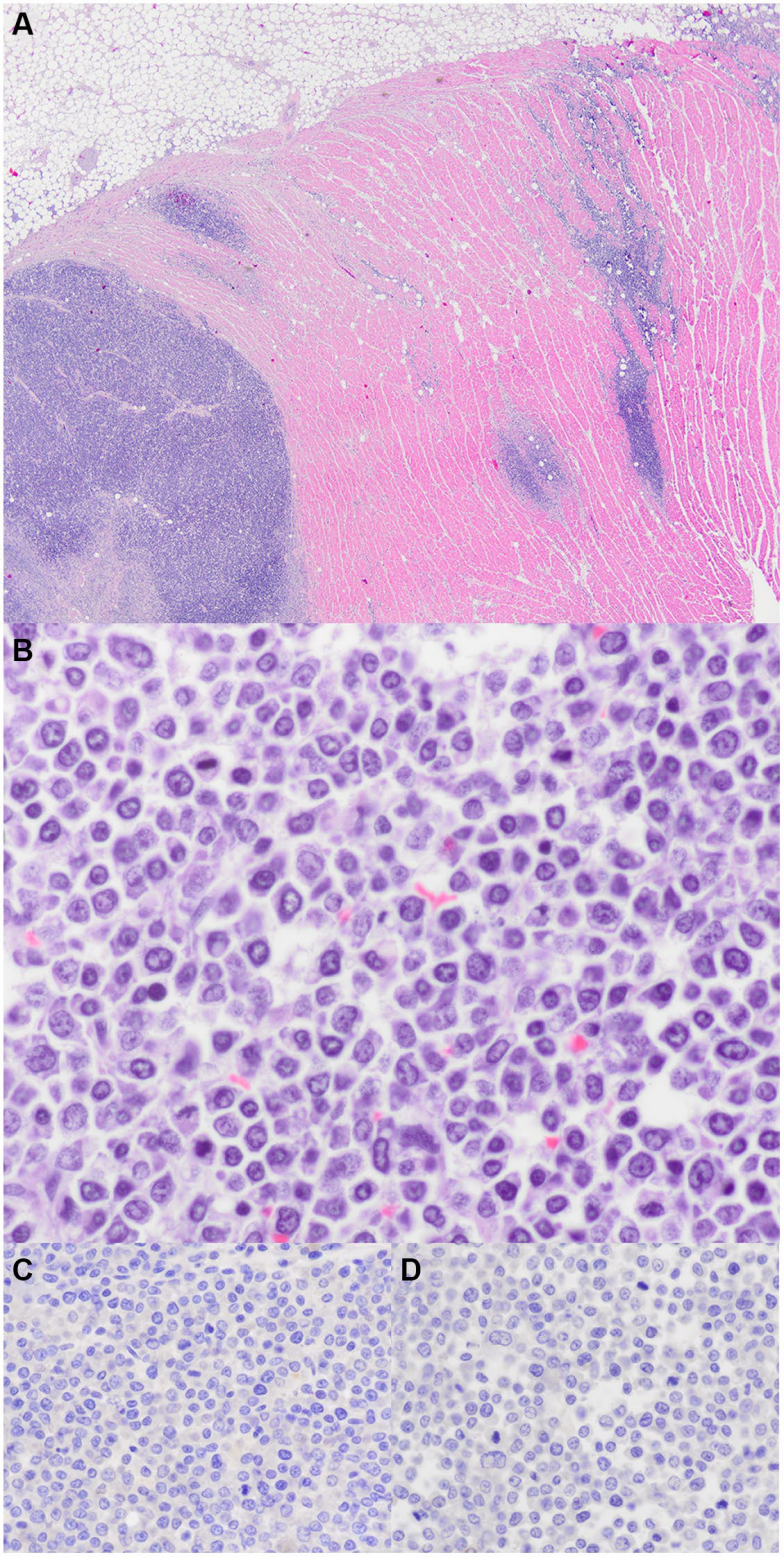
Myocardial lymphoma. **(A)** Right ventricular outflow tract. The myocardium is multifocally expanded by sheets of neoplastic round cells (H&E stain, 20x). **(B)** Neoplastic round cells have scattered mitoses (H&E stain, 600x). **(C)** Immunohistochemistry for CD3; no immunoreactivity (600x). **(D)** Immunohistochemistry for CD8; no immunoreactivity (600x).

**Figure 4:**
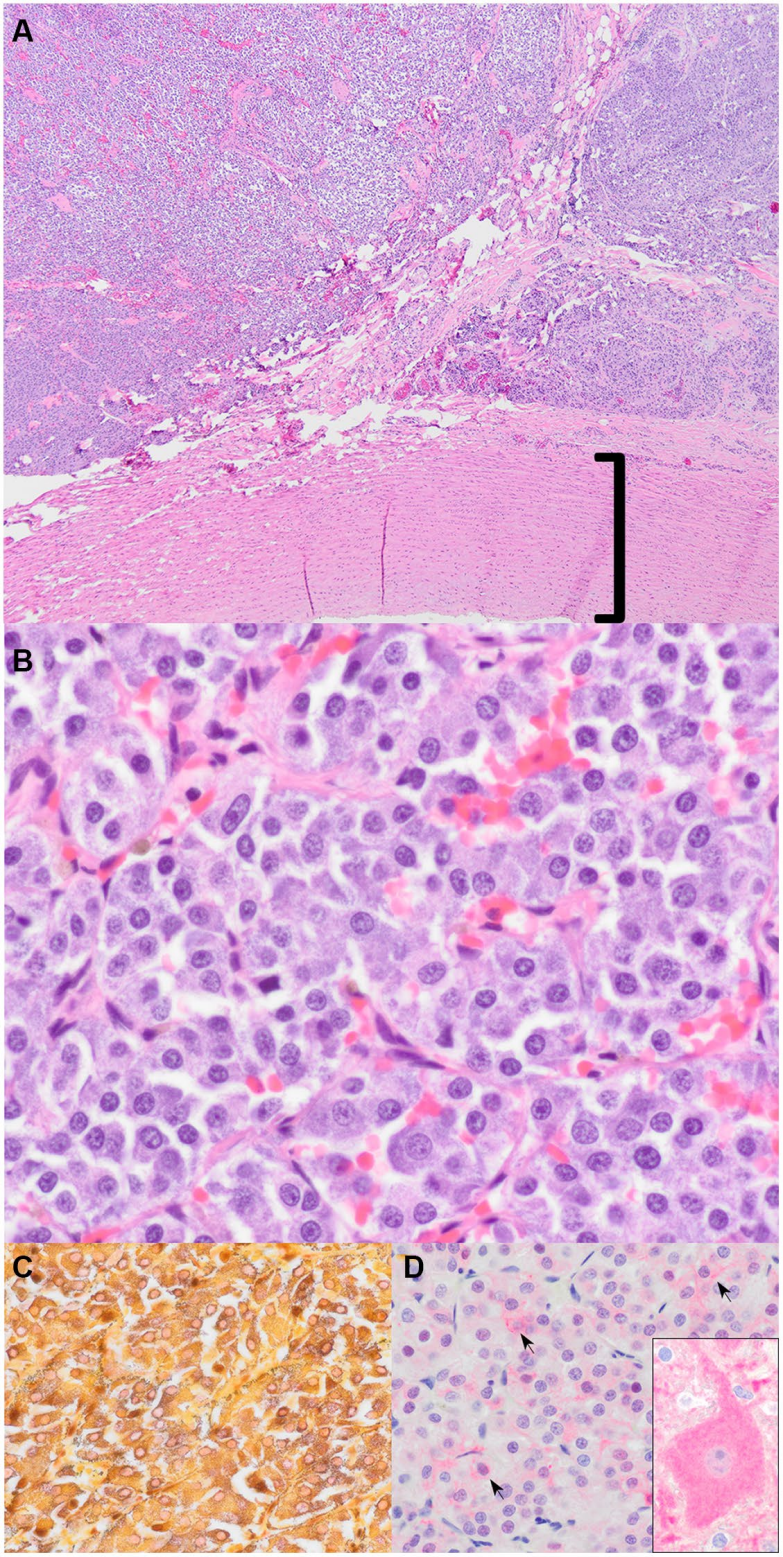
Aortic chemodectoma. **(A)** Mass adjacent to the aorta (bracket) (H&E stain, 40x). **(B)** Nests and packets of polygonal neoplastic cells with granular basophilic cytoplasm (H&E stain, 400x). **(C)** Churukian–Schenk stain highlights the argyrophilic (black) neurosecretory granules in the neoplastic cells (H&E stain, 600x). **(D)** Immunohistochemistry for neuron-specific enolase (NSE). There is faint pink cytoplasmic and membranous immunoreactivity (black arrows indicate some of the immunoreactive cells) (600x). The inset is positive control brain tissue with a large immunoreactive pyramidal neuronal cell body (600x).

The myocardial neoplastic round cells did not contain metachromatic granules when stained with Toluidine blue. Additionally, these cells were not immunoreactive to CD3 (T-cell), CD79 (B-cell), or CD20 (B-cell) but were surrounded by moderate numbers of CD3 immunoreactive cells. Neither the myocardial neoplastic round cells nor the chemodectoma cells were immunoreactive to Mum-1 (plasma cell), CD117 (c-kit; mast cell), or CD18 (histiocyte). There were CD18 immunoreactive cells scattered around both populations of neoplastic cells. The chemodectoma cells, but not the neoplastic round cells, were immunoreactive to neuron-specific enolase (NSE). Figures 3, 4 highlight the immunoreactivity of the two tumors.

## Discussion

This case describes a middle-aged dog with a rare cardiac tumor phenotype of the right ventricular myocardium that presented with signs of RHF. The case serves to emphasize the importance of considering cardiac lymphoma as a differential diagnosis, although uncommon in this species. In a case series of 12 dogs diagnosed with cardiac lymphoma, all but one dog was a large breed with a wide age range (2 to 16 years). A rare case of cardiac lymphoma clustering has been described in a family of Otterhounds, with the sire and two sibling offspring affected between 5 and 6 years of age; a genetic or viral factor has been proposed (9). No information was available about related animals to the dog in our case report. The RHF signs were likely secondary to the chronic effusion into the pericardial space and the myocardial infiltrates, which may have contributed to reduced right ventricular compliance. In the case series, clinical signs developed a median of 3 days prior to presentation, and most commonly, they were lethargy and dyspnea. Right-sided heart failure is described as the most common presenting clinical finding in canine cardiac lymphoma, as is evident from the following descriptions: 8 of 12 dogs in one case series presented with ascites; a 9-year-old Cocker spaniel with a large right intra-atrial mass presented with ascites, hepatomegaly, and distended caudal vena cava; and a 10-week-old puppy presented with pleural and peritoneal effusion (2, 10, 11). Right heart failure is also the most common presenting clinical finding in human cardiac lymphoma (5).

The only abnormal structural echocardiographic finding in 9 of the 12 dogs in which echocardiography was performed was a pericardial effusion without visible masses in the myocardium. Other case reports have described irregular thickening and heterogenous echodensities in the ventricular myocardium (11–13). Specifically, echocardiographic features, if present, include heterogeneously echodense mass (13), thickened interventricular septum (12), and thickening and irregularities of the left ventricular wall and septum in three related otterhounds (9), and in a 10-week-old puppy, a moderately dilated and hypokinetic ventricle (11). The antemortem diagnosis of left ventricular wall thickening was later confirmed on necropsy as a result of myocardial lymphoma that had also infiltrated the left ventricle. Abnormal echotexture of the left ventricle was not recognized on the echocardiogram, and infiltration with neoplasia in this chamber was overlooked. This highlights the low sensitivity of echocardiography in screening for myocardial tumors.

Knowledge of canine cardiac tumor phenotype makes echocardiography the diagnostic test of choice for investigating the etiology of pericardial effusion; however, it is considered moderately accurate, with 86% agreement with location and only 65% agreement on tissue type diagnosis when compared to necropsy (14). The presence of pericardial effusion improves the accuracy of echocardiography, as anechoic pericardial fluid is able to provide contrast to cardiac tumors, with a reported sensitivity of 82% and a specificity of 100% (3). Even though cardiac magnetic resonance imaging (cMRI) is considered the gold standard for diagnosing cardiac neoplasia in humans, in a small study where cMRI examined dogs with pericardial effusion, this modality did not demonstrate a diagnostic advantage. In many cases, the results were discordant, and the results were influenced by the experience of the radiologist (15). Potentially, transesophageal echocardiography or the use of echocardiogram contrast agents may offer greater diagnostic utility than standard transthoracic echocardiography, but this comparison has not been made in dogs. Surprisingly, pericardial effusion is not a common complication of cardiac tumors, present in only 16% of all dogs diagnosed with cardiac tumors on necropsy. This is in contrast to the higher prevalence of concurrent pericardial effusion in 42% of dogs with echocardiographic detection and later confirmed (on necropsy) diagnosis (14, 16). The benefit of this imaging modality is the information provided on the mobility, infiltrative nature, location of attachment, and hemodynamic consequences of a cardiac mass. Despite the high specificity quoted, differential diagnoses should include infectious vegetation, sterile thrombi, and myocardial hematomas. The referenced sensitivity highlights that an inherent risk of false negative echocardiographic diagnoses exists, particularly in the absence of pericardial effusion, as highlighted by the canine lymphoma study that was unable to detect a cardiac mass in all dogs that underwent echocardiography (2).

Rarely, arrhythmia detection is the reason for an echocardiogram and the detection of a myocardial mass. Sinus rhythm was recorded in all 12 dogs in the case series, in addition to one dog having an intermittent atrioventricular block (AV) (2). Several individual case reports have described complete AV block and ventricular ectopy associated with extranodal cardiac lymphoma (11–13). Other arrhythmia included ventricular tachycardia in the 10-week-old puppy (11), one of the 12 dogs with myocardial lymphoma having accelerated idioventricular rhythm and ventricular premature complexes (2), one of the 3 related otterhounds all diagnosed with cardiac lymphoma had reported ventricular ectopy (9), and one dog with sinus rhythm and intermittent accelerated idioventricular rhythm and premature ventricular complexes at the time of presentation, which later died 2 h after pericardiocentesis due to ventricular fibrillation (12).

Of the 12 dogs described in the largest case series, 5 were treated with various combinations of multiagent chemotherapy, either after therapeutic pericardiocentesis or pericardiectomy Median survival time was 157 days, which was significantly longer than dogs that did not receive chemotherapy, reported as 7 days (2). No other factors were found to be prognostic of an unfavorable outcome in this study. A case report described a favorable response of a myocardial lymphoma to prednisolone with a relapse 1 year later (9). The dog in our case report was euthanized after echocardiography results were discussed with the owner. It is unknown if the presence of effusions and arrhythmia or the high-grade null-cell type would have contributed to the overall outcome if the owners had elected therapy.

The immunohistochemistry of canine cardiac lymphoma has been described in the veterinary literature. Cardiac lymphoma immunoreactive to CD3, CD79a, and CD18 was confirmed in the dog with an AV block (13); co-expression of CD3 and CD20 and molecular clonality consistent with clonal T-cell receptor gamma-rearrangement in an 8-year-old pug with peripheral nerve sheath and cardiac lymphoma (17); a T-cell lymphoma (immunoreactive to CD3 but not CD79) in a 9-year-old labrador with diffuse myocardial thickening and pericardial effusion; and 3 of the 12 dogs in the canine lymphoma case series that had staining performed, found 2 were consistent with T-cell and one with B-cell lymphoma (2). The findings of the microscopic examination and immunohistochemistry in this case report confirmed the presence of a chemodectoma located near the aortic root and a separate neoplastic round cell population. The histologic features of the round cells were consistent with a small cell, high-grade, lymphoma; the grade designation was based on the mitotic count (18). The size of the neoplastic cells was listed according to the description from Valli et al. (19). Immunohistochemistry revealed that the neoplastic round cells were not immunoreactive to leukocyte markers for B-cells, T-cells, plasma cells, histiocytes, or mast cells, and thus, the diagnosis of a null-cell lymphoma was made. Less than 1% of canine lymphomas are null-cell type (lacking immunoreactivity to CD3, CD79a, CD4, and CD8), and these may represent NK cell neoplasms; however, the immunohistochemical markers and case definitions for natural killer (NK) cell lymphomas in veterinary medicine have not yet been determined (20). A morphological study of 608 cases of canine lymphoma described null-cell lymphoma as CD3 and CD79a negative, and these comprised 0.8% of all lymphoma cases (5 of 608) (21). The description included pleomorphic mixed lymphomas in four dogs and a large-cell lymphoma in another. Null-cell lymphoma is poorly characterized in dogs due to the lack of a canine CD56 antibody, which is a marker of NK lymphoma (21, 22). The human extranodal NK/T-cell lymphoma is an aggressive systemic proliferation of NK or NK-like T-cells the predilection of the nasal, nasopharynx, gastro-intestinal tissue, and testis (22). The cancer is characterized by rapid, multiple, extranodal involvement, with frequent hemophagocytic syndrome, and multi-organ dysfunction. It was suspected in two dogs with large granular lymphoma and hemophagocytic syndrome.

## Conclusion

Null-cell-type lymphoma is a rare cardiac tumor that manifests in the acute onset of RHF in a middle-aged dog and should be considered a differential diagnosis when echocardiography detects wall thickening and myocardial nodules. The prognosis for this cell type is unknown in the dog, but the human counterpart is considered aggressive.

## Data availability statement

The original contributions presented in the study are included in the article/supplementary material, further inquiries can be directed to the corresponding author.

## Ethics statement

Ethical approval was not required for the studies involving animals in accordance with the local legislation and institutional requirements because IACUC approval is not needed if a sample is collected from a necropsy case and client approval is given. Written informed consent was obtained from the owners for the participation of their animals in this study.

## Author contributions

LK: Conceptualization, Investigation, Writing – original draft. KN: Conceptualization, Investigation, Writing – review & editing. PK: Conceptualization, Investigation, Writing – review & editing.
